# Oxidative Damage and Telomere Length as Markers of Lung Cancer Development among Chronic Obstructive Pulmonary Disease (COPD) Smokers

**DOI:** 10.3390/antiox13020156

**Published:** 2024-01-26

**Authors:** Elizabeth Córdoba-Lanús, Luis M. Montuenga, Angélica Domínguez-de-Barros, Alexis Oliva, Delia Mayato, Ana Remírez-Sanz, Francisca Gonzalvo, Bartolomé Celli, Javier J. Zulueta, Ciro Casanova

**Affiliations:** 1 Department of Internal Medicine, Dermatology and Psychiatry, University of La Laguna, 38296 San Cristóbal de La Laguna, Spain; casanovaciro@gmail.com; 2 Instituto de Enfermedades Tropicales y Salud Pública de Canarias (IUETSPC), 38029 San Cristóbal de La Laguna, Spain; angelica4arealejos@gmail.com (A.D.-d.-B.); amoliva@ull.edu.es (A.O.); 3 Centro de Investigación Biomédica en Red de Enfermedades Infecciosas (CIBERINFEC), Instituto de Salud Carlos III, 28029 Madrid, Spain; 4 Department of Pathology, Anatomy and Physiology, School of Medicine and Sciences, University of Navarra, 31008 Pamplona, Spain; lmontuenga@unav.es; 5 CIMA—Centro de Investigación Médica Aplicada, University of Navarra, 31008 Pamplona, Spain; aremirez@unav.es; 6 Navarra Institute for Health Research (IdISNA), 31008 Pamplona, Spain; 7 Centro de Investigación Biomédica en Red de Cáncer (CIBERONC), 28029 Madrid, Spain; 8 Department of Pharmaceutical Technology, University of La Laguna, 38296 San Cristóbal de La Laguna, Spain; 9 Pulmonary Division, Research Unit, Hospital Universitario Nuestra Señora de Candelaria, 38010 Santa Cruz de Tenerife, Spain; deliamayato@gmail.com (D.M.); franciscagonzalvo@hotmail.com (F.G.); 10 Pulmonary Critical Care Medicine Division, Brigham and Women’s Hospital, Harvard Medical School, Boston, MA 02115, USA; bcelli@copdnet.org; 11 Pulmonary, Critical Care and Sleep Medicine Division, Mount Sinai Morningside Hospital, Icahn School of Medicine, New York, NY 10025, USA; javier.zulueta@mountsinai.org; 12 Centro de Investigación Biomédica en Red de Enfermedades Respiratorias (CIBERES), Instituto de Salud Carlos III, 28029 Madrid, Spain

**Keywords:** lung cancer, COPD, telomeres, oxidative stress, 8-OHdG, biomarkers

## Abstract

Lung cancer (LC) constitutes an important cause of death among patients with Chronic Obstructive Pulmonary Disease (COPD). Both diseases may share pathobiological mechanisms related to oxidative damage and cellular senescence. In this study, the potential value of leucocyte telomere length, a hallmark of aging, and 8-OHdG concentrations, indicative of oxidative DNA damage, as risk biomarkers of LC was evaluated in COPD patients three years prior to LC diagnosis. Relative telomere length measured using qPCR and serum levels of 8-OHdG were determined at the baseline in 99 COPD smokers (33 with LC and 66 age-matched COPD without LC as controls). Of these, 21 COPD with LC and 42 controls had the biomarkers measured 3 years before. Single nucleotide variants (SNVs) in TERT, RTEL, and NAF1 genes were also determined. COPD cases were evaluated, which showed greater telomere length (*p* < 0.001) and increased serum 8-OHdG levels (*p* = 0.004) three years prior to LC diagnosis compared to the controls. This relationship was confirmed at the time of LC diagnosis. No significant association was found between the studied SNVs in cases vs. controls. In conclusion, this preliminary study shows that longer leucocyte telomere length and increased 8-OHdG serum levels can be useful as early biomarkers of the risk for future lung cancer development among COPD patients.

## 1. Introduction

Chronic obstructive pulmonary disease (COPD) and lung cancer (LC) are major public health problems, being the third- and fifth-leading causes of death worldwide [1]. Both diseases are thought to result from an interaction between the genome and the surrounding environment [2]. There is significant evidence that an enhanced inflammatory response due to noxious particles and gases contributes to the frequent association between COPD and LC [3]. Tobacco exposure is the most common risk factor for COPD and LC, with lung cancer incidence being 2–5 times that of smokers without COPD [3,4]. LC also constitutes an important cause of death among patients with COPD [5]. This suggests that both diseases may share mechanisms, with oxidative stress playing a central pathobiological role [2,6]. Oxidative stress leads to DNA damage and impairs an adequate repair process, leading to cellular senescence [7]. This suggests that changes in oxidative stress and senescence markers could help to define the pathobiological processes at an earlier stage of LC development.

Telomere shortening is frequently used as a biomarker of cellular senescence. The main function of telomeres is to protect chromosomes from end-to-end fusions, misrepair, and degradation [8]. Telomeres shorten with each cell division until they reach a critically short length when they trigger apoptosis or cellular senescence [9]. Contradictory results relate telomere length and LC in studies that measured telomere length at the time of LC diagnosis; some studies found shorter telomeres in relation to LC [10,11], while others have reported longer telomeres [12,13,14]. However, few studies have evaluated the relationship between telomere length and LC risk before diagnosis. Doherty et al. found that short telomere length was associated with an increased risk of death from cancer [11], while Shen et al. reported longer telomeres in relation to LC risk in the peripheral white blood cells of male smokers [12]. No study has investigated the dynamic change of telomere length over time in relation to LC development among patients with COPD.

Telomerase reverse transcriptase (TERT) encodes the catalytic subunit of telomerase, an enzyme that maintains telomere ends while acting with an RNA component (TERC) in the process of adding telomeric nucleotide repeats (TTAGGG). Telomerase expression is higher in progenitor and cancer cells in contrast with the absent or low levels found in normal somatic cells [15]. Multiple genetic and epigenetic mechanisms are proposed to be involved in the telomerase reactivation that occurs in relation to oncogenesis [16]. RTEL1 (Regulator of Telomere Length 1) is an essential helicase required for telomere integrity; it has multiple roles in the control and maintenance of telomere length and sites of mitotic DNA damage, being involved in the repair of DSBs (double strands breaks). It was also reported to have a tumor-suppressive function [17]. Nuclear assembly factor 1 (NAF1) is another key factor in telomerase biogenesis [15].

A few single-nucleotide variants (SNVs) in *TERT*, *RTEL1*, and *NAF1* genes are reported to associate with long telomeres [18,19], suggesting that they may be associated with an increased risk of cancer, as shown for rs2736100 in the *TERT* gene that is associated with an increased risk of adenocarcinoma [19].

On the other hand, other markers related to oxidative damage could help characterize this scenario. Aoshiba et al. (2012) suggested that the DNA damage caused by long-term smoking accumulates over the years before COPD and lung cancer develop [20]. Oxidative stress may damage DNA in different forms, as is the case for the change of genomic bases to species like 8-hydroxy-2′-deoxyguanosine (8-OHdG), which leads to DNA hypomethylation and subsequent genomic instability [21]. In this line, higher concentrations of 8-OHdG were reported in the lung tissue and peripheral blood mononuclear cells of COPD smokers [22]. Although increased levels of 8-OHdG are associated with cancers such as hepatocellular carcinoma, breast cancer, colorectal cancer, and prostate cancer [23], there are still controversies regarding this marker. To our knowledge, 8-OHdG as a potential non-invasive biomarker of LC risk in COPD patients, before LC diagnosis, has not been explored.

We hypothesized that leucocyte telomere length and serum levels of 8-OHdG could be risk markers of LC in smoker patients with COPD. We prospectively evaluated the potential value of these markers in COPD patients three years prior to LC diagnosis compared to COPD smokers without LC during the same observation period, with the latter group serving as controls. Secondary single nucleotide polymorphisms in the *TERT*, *RTEL1*, and *NAF1* genes were analyzed in relation to telomere length.

## 2. Materials and Methods

### 2.1. Subjects

**Figure 1:**
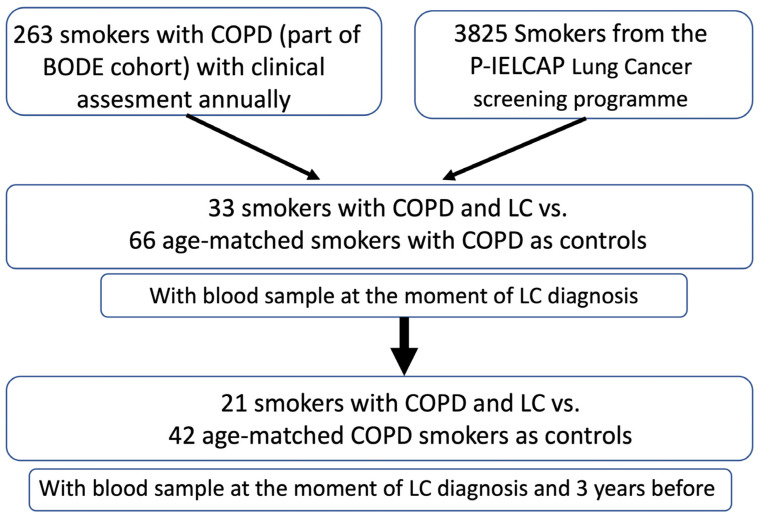
Consort diagram illustrating the selection and assessment of patients and controls included in this study.

A total of 263 smokers with a COPD diagnosis were included in this study. The participants belonged to two cohorts: 1—patients screened at the Hospital Universitario La Candelaria, Tenerife, Spain (Tenerife-cohort), part of the BODE cohort, with an annual follow-up as detailed previously [24]; and 2—patients screened at the P-IELCAP, Pamplona-International early-detection program (3825 individuals) [25]. Of these patients, 33 developed LC during the mean 72 months of follow-up and were enrolled in the study (13 in Tenerife and 20 in the Pamplona cohort). Of these patients, 21 had blood samples obtained at two time points—at LC diagnosis and 3 years before. The latter time point was set up to coincide with sampling and as a point close to the time of diagnosis but early enough to anticipate the development and progression of cancer. As controls, we included 66 COPD patients who did not develop LC during the follow-up period and who were age- and gender-matched with cases (Figure 1).

Briefly, patient inclusion criteria were age, >40 years; smoking history, >15 pack-years; and post-bronchodilator FEV_1_/FVC ratio, <0.70. Patients were clinically stable for at least 6 weeks at the time of evaluation. Pulmonary function, measured according to ATS-ERS guidelines [26,27], dyspnea [28], the BODE Index [29], and the Charlson Index [30], was measured annually. All-cause mortality was recorded. Patients received inhaled respiratory therapy as recommended by the international guidelines [26,27]. The presence of emphysema at the baseline was determined using criteria established by the Fleischner Society [31]. Patients were excluded from the study if they had uncontrolled co-morbidities such as malignancy at the baseline, asthma, pulmonary fibrosis, or pulmonary diseases other than COPD.

The study was approved by the institutional review board of HUNSC (PI14/12). All participants provided written informed consent.

### 2.2. Telomere Length Measurement

DNA was extracted from whole blood using the QIAamp DNA Mini Kit (GE Healthcare, Chicago, IL, USA). Telomere length and albumin (reference gene) were measured in triplicate in each sample using a qPCR-based protocol as described previously [32]. Two control DNA samples were assayed per run as a normalizing factor. Also, calibrator samples were assayed on each PCR plate to control for inter-plate and intra-plate coefficients of variance (CV).

Telomere length was calculated as a ratio of telomere to albumin. The T/S ratio for an experimental DNA sample is T (the number of nanograms of the standard DNA that matches the experimental sample for the copy number of the telomere template) divided by S (the number of nanograms of the standard DNA that matches the experimental sample for copy number of the albumin single-copy gene) [33]. T/S ratios were calculated using the “∆∆Cp with efficiency correction” method [34]. Conversion of the T/S ratio to base pair was calculated for every subject based on the equation y = 1114.58 + 10,373.13 × x of the correlation analysis, where x is the T/S ratio as previously determined [32].

### 2.3. Measurement of 8-Hydroxy-2′Deoxyguanosine (8-OHdG) in Serum

Serum/plasma concentrations of 8-OHdG were determined using a commercial sandwich enzyme-linked immunosorbent assay (ELISA) kit—STA-320 OxiSelect™ Oxidative DNA Damage ELISA Kit (CellsBiolab, Inc. San Diego, CA, USA). Absorbance was read on a spectrophotometer (EnSpire Perkin Elmer, PerkinElmer, Inc., Waltham, MA, USA) using 450 nm as the primary wavelength.

### 2.4. TERT, RTEL, and NAF SNV Genotyping

Genotyping was performed on genomic blood DNA samples obtained from the studied patients included in this study. The SNVs were assessed using Taqman^®^ assays (*TERT* A__rs2736100_C, *RTEL1* A___rs755017_G, and *NAF1* A___rs7675998_G) (ThermoFisher Scientific, Inc., Waltham, MA, USA) employing a Quant 5™ Real-Time PCR System (ThermoFisher Scientific, Inc., Waltham, MA, USA). The SNVs were selected according to a review of the scientific literature regarding their influence on telomere length, the minor allele frequencies of the variants in European populations, and the availability of genotyping methodologies.

### 2.5. Statistical Analysis

For characterizing the sample, we used the following summary statistics: relative frequency of each category, 50th percentiles (5–95th), and non-scale normal and mean ± SD, as appropriate. The comparison between cases and controls was carried out using Pearson’s Chi-square, Mann–Whitney U, Wilcoxon, ANOVA, Fisher Exact, and Kruskal–Wallis tests. The correlations between variables were estimated using the Spearman or Pearson correlation tests. Relative telomere length (T/S) was inversely correlated with age, so all subsequent analyses were adjusted by this variable. The biomarker analysis was performed at two time points—at the point of LC diagnosis and 3 years before (median follow-up = 36 months).

Cases and controls were age- and gender-matched. Using the Cox multivariate analysis, the following covariates were included: smoking status (pack years), BMI, Kco, the Charlson Index, and the presence of emphysema (based on a visual score using CT scans). The sensitivity and specificity of relative telomere length (T/S) and 8-OHdG levels for distinguishing those patients who developed lung cancer early were determined by receiver operating characteristic (ROC) curves. The associations between SNPs and clinical data were tested using unconditional logistic regression while adjusting for sex and pack years smoked. The Hardy–Weinberg equilibrium was tested for each of the SNPs using Fisher’s exact test. Haplotype blocks were constructed using Haploview v.4.1 [35] and SNPSTAT software (https://www.snpstats.net/start.htm) [36].

SPSS 26.0 IBM Co and R software (https://www.r-project.org/) were used for all statistical analyses, and two-tailed *p*-values < 0.05 were considered significant.

## 3. Results

### 3.1. Baseline Findings

The clinical characteristics of the 33 patients with COPD who developed LC, and the 66 controls with COPD and no cancer, are shown in Table 1.

Patients had a mean age of 63 years, were mainly men (85%), and included heavy smokers, although the proportion of active smokers was slightly lower in the COPD patients who developed LC. Most of the COPD patients (90%) had moderate airway obstruction. Smokers with COPD and LC presented higher FEV_1_ and lower K_CO_ values but a similar percentage of emphysema as visualized using CT scans than the COPD controls. Non-small cell lung cancer (NSCLC) was the primary LC histological subtype, as follows: 61% adenocarcinoma, 27% squamous carcinoma, 3% microcytic carcinoma, and two cases of undifferentiated carcinoma.

### 3.2. Changes over Time

The clinical characteristics of the 21 COPD patients who developed LC and the 42 who did not and had the biomarker measurements are shown in Table 2 and Table S1. For similar age and gender, patients who developed LC had higher FEV_1_ but slightly lower K_CO_ values, with all other parameters being similar.

**Figure 2:**
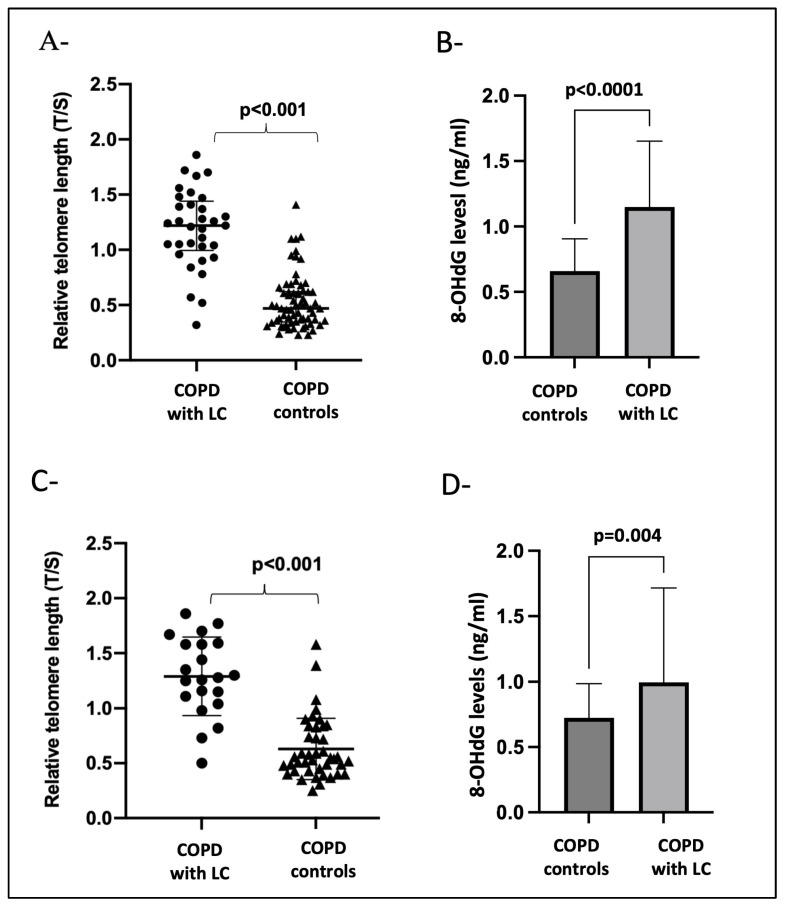
Panels (**A**,**B**) show the biomarker differences between COPD-with-LC patients (n = 33) at the time of diagnosis and age- and gender-matched COPD controls (n = 66). (**A**) T/S, leukocyte relative telomere length; (**B**) 8-OHdG levels in serum. Panels (**C**,**D**) show the biomarker differences between COPD patients with LC (n = 21) three years before diagnosis versus COPD patients (n = 42) that did not develop LC during follow-up monitoring. (**C**) T/S, leukocyte relative telomere length; (**D**) 8-OHdG levels in serum. A *p*-value < 0.05 was considered significant.

At the time of LC diagnosis and after adjusting for age, pack years of smoking, BMI, and emphysema (Figure 2A,B), the patients with COPD that developed LC had longer telomeres (*p* < 0.001) and increased 8-OHdG levels (*p* < 0.0001) compared with the COPD cases without LC. Importantly, longer telomere length (*p* < 0.001) and increased 8-OHdG levels (*p* = 0.004) could be detected three years before LC diagnosis when compared to the COPD controls who did not develop LC (Figure 2C,D).

An increased risk of developing LC in COPD patients was found in relation to telomere length (T/S) and 8-OHdG levels independently of smoking habit, BMI, or the presence of emphysema (Table 3). The combined effect of telomere length (T/S) and 8-OHdG levels did not significantly increase the risk.

**Figure 3:**
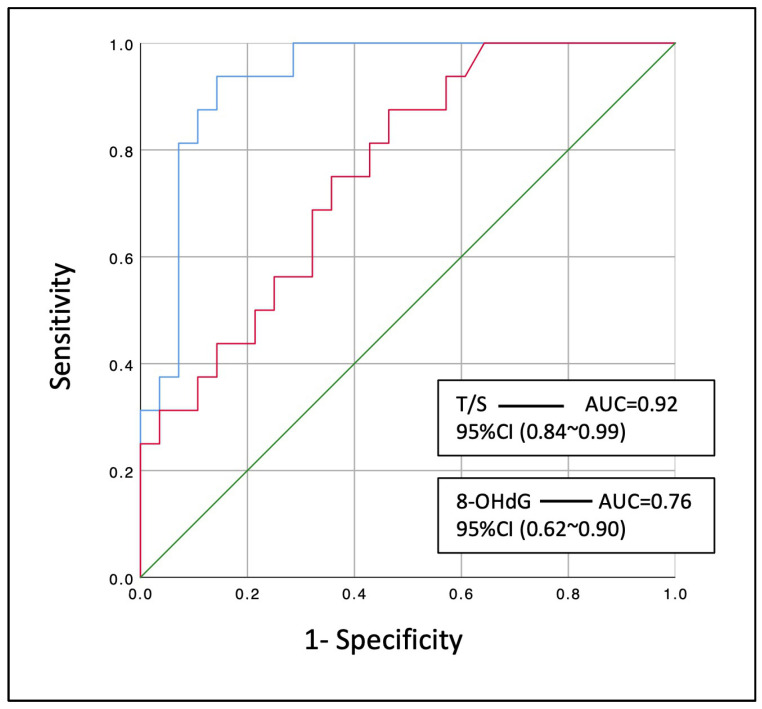
Diagnostic performance of leucocyte relative telomere length (T/S) and serum 8-OHdG at discriminating smoking COPD patients that developed lung cancer within the next three years. Blue line: T/S, relative telomere length; Red line: 8-OHdG levels; AUC: area under the curve; 95% CI: 95% confidence interval.

The sensitivity analysis revealed that relative telomere length (T/S) [AUC: 0.92 (95% CI: 0.84–0.99), *p* < 0.001] could significantly discriminate those smokers with COPD that developed LC within the next three years from the control smokers that did not (Figure 3). In the same way, the marker of oxidative DNA damage (8-OHdG) [AUC: 0.76 (95% CI: 0.62~0.90), *p* = 0.004] could modestly discriminate these patients from the COPD controls.

### 3.3. TERT, RTEL1, and NAF1 Single Nucleotide Variants

The allele and genotype frequencies of *TERT*, *RTEL1*, and *NAF1* SNVs are presented in Table 4. The genotypic frequencies of all gene single-nucleotide variants were accomplished with Hardy–Weinberg equilibrium. There were no significant differences in the genotype frequencies between the COPD-with-LC and COPD control groups (*p* > 0.05), even after correction for multiple comparisons. Global haplotypes (C-A-G; *TERT–RTEL1-NAF1*) did not differ in their frequency distribution between the analyzed groups (controls = 0.4223 vs. cases = 0.3125; *p* = 0.9).

## 4. Discussion

To our knowledge, this is the first prospective study exploring the relationships between leukocyte telomere length, serum/plasma concentrations of 8-OHdG, and LC risk in COPD patients 3 years prior to LC diagnosis. This preliminary study has several important findings: First, longer leucocyte telomeres were observed in COPD patients prior to the diagnosis of LC when compared to age-matched COPD patients who did not develop LC during the same period of observation. Second, increased levels of 8-OHdG were detected in the serum/plasma of the COPD patients who developed LC three years prior to diagnosis.

### 4.1. Telomere Length

In human somatic cells, telomeres progressively shorten at a rate of 50–200 bp with each cell division [37]. In previous studies, we found accelerated telomere shortening in COPD patients followed for 10 years (183 bp per year) compared to smoking controls of the same age and sex and without COPD [32].

In this study, we found that COPD patients who developed LC during follow-up monitoring had longer telomeres than individuals with COPD who did not. Telomeres are thought to be involved in the initiation and progression of cancers [38]. Telomerase activity can promote tumor development by ensuring the maintenance of telomere length above a critical short threshold to prevent senescence or apoptosis [39], although this may also increase the risk of acquiring genetic abnormalities. Telomerase is aberrantly expressed in more than 90% of human cancers [40]. In addition to this, there is another mechanism for telomere elongation, known as “alternative telomere lengthening” or ALT, which has been documented in 10–15% of cancers [41].

Many studies have reported an association between increased telomere length and the risk of various cancers, including melanoma, basal cell carcinoma, glioma, lymphoma, urogenital cancer, and lung cancer [42,43,44]. These results are supported by a genetic basis through studies, where certain gene variants modulate telomere length, with alleles segregating with long telomeres associated with the increased risk of different diseases. This is the case for SNVs near the *TERT*, *RTEL1*, and *NAF1* genes, which show differential effects on telomere length. For these SNVs, one of the alleles that promotes short telomeres strongly associates with idiopathic pulmonary fibrosis, while the other long-telomere alleles associate with LC [44]. However, none of the studied alleles at these variants are significantly associated with lung cancer, at least in the studied COPD cohort. Further studies regarding genetics and epigenetic mechanisms are needed to elucidate these findings. In our study, longer telomeres maintained their length for years in individuals with COPD who developed LC during follow-up monitoring. Our results are supported by a metanalysis from three cohorts of smokers and non-smokers, where a significant association between longer leukocyte telomere length and risk of LC—particularly adenocarcinoma—was found [45]. Our findings agree with other studies that reported an association between longer leucocyte telomeres and LC [13,14] independently of the presence of COPD or emphysema [14] but, importantly, extends across previous cross-sectional research when evaluating longitudinal potential markers of LC risk as early as 3 years prior to diagnosis. Our group previously evaluated a potential profile of microRNAs as risk markers of cancer development and found a signature of two miRNAs (miR-1246 and miR-206) with a moderate capacity of detection [46]. This new marker has a strong capacity to discriminate those patients with COPD who are at increased risk of developing lung cancer.

### 4.2. 8-OHdG Serum/Plasma Levels

Oxidative DNA damage (8-OHdG formation), along with the impaired induction of hOGG1, has been studied in the lungs of COPD patients. Increased 8-OHdG levels in DNA from peripheral blood were reported to be associated with poor lung function in smoking COPD patients in contrast to hOGG1 induction [22]. Another study observed that urinary excretion levels of 8-OHdG were significantly higher in smokers than in subjects who had never smoked, confirming oxidative damage. Importantly, these levels were also found to be significantly increased in lung cancer patients, although decreasing with a higher severity of disease [47]. An et al. (2019) found that the expression of 8-OHdG by immunohistochemistry in non-small cell lung cancer (NSCLC) tissue was associated with a good prognosis (never smoked, low T category, or negative lymph node) [48]. All these results highlight the importance of studying this biomarker longitudinally in well-characterized cohorts to unravel possible controversies. Our study extends these observations by showing higher levels of 8-OHdG 3 years before the diagnosis of LC, further strengthening the association of this biomarker with COPD smokers and subsequent LC development.

Our study has some limitations. Firstly, and perhaps most importantly, the sample size may be considered small compared to some cross-sectional studies. However, this may not hold for a prospective study like ours, where a large, very well-characterized cohort of COPD patients was included and who were followed for a long period, thereby allowing the monitoring of the development and diagnosis of LC. Secondly, telomere length was measured in leucocytes and, therefore, our findings may not reflect the process occurring in the whole lung. It is already known that telomere length in blood correlates well with telomere length in the lung tissue of COPD patients [49], with similar rates of shortening between different tissues [50]. In addition, blood has the advantage of being a non-invasive, easy-to-access biological sample that allows the analysis of large cohorts of individuals. Third, we did not investigate other markers of aging and DNA damage alongside telomere length and 8-OHdG. Current evidence indicates that COPD may be due to accelerated lung aging, where accumulations of senescent cells persist in the lung tissue and release multiple inflammatory mediators and ROS [51]. The measurement of markers of immunosenescence could help to deeply characterize this complex scenario and its impact and contribution to immunosenescence, oxidative balance, and related inflammaging in COPD patients in relation to LC development. Lastly, we are aware that pharmacological treatment is an important factor that may alter the oxidative balance; however, the patients included in this study only received inhaled respiratory therapy as recommended by international guidelines, and none of these treatments have been shown to effectively modify the oxidative balance [52].

On the other hand, this study has several strengths, including the fact that the COPD patients proceeded from two different centers, with the biomarker measures that precede the diagnosis of LC identified in both samples. Finally, these findings provide a new perspective on LC screening. Until now, only the use of low-dose computed tomography (LDCT) screening has been shown to improve survival in older smoking subjects [53]. The addition of complementary biomarkers to other clinical data may help refine the risk-stratification process of cancer screening for subjects at risk of LC development [54].

In conclusion, our results suggest that longer leucocyte telomere length and increased 8-OHdG serum levels may serve as early biological markers of future lung cancer development among COPD patients, which can be employed along with traditional methods to help define LC risk during the screening of patients.

## Figures and Tables

**Table 1:** Demographical and clinical characteristics at the baseline of COPD patients who developed lung cancer (LC) during follow-up monitoring and those who did not.

Variable	COPD with LC n = 33	COPD without LC n = 66	*p*-Value
Age *	63 ± 9	63 ± 9	-
Sex (male%)	85	85	-
BMI *	28 ± 5	28 ± 5	0.719
Smoking habit (pack-year) ‡	64 ± 24	61 ± 27	0.631
Active smoker (%)	50	70	0.218
FEV_1_ (L) *	2.24 ± 0.74	1.67 ± 0.72	**0.001**
FEV_1_ (% pred) *	79 ± 21	62 ± 26	**0.001**
FVC (% pred) *	105 ± 20	91 ± 24	**0.005**
FEV_1_/FVC (% pred) *	58 ± 11	52 ± 14	**0.018**
PaO_2_ *§	72 ± 6	71 ± 12	0.872
K_CO_ *§	68 ± 25	84 ± 27	0.084
IC/TLC (%) *§	34 ± 8	34 ± 9	0.881
6MWD (mts) *§	486 ± 115	497 ± 101	0.747
Dyspnea mMRC **	0 (0–1)	1 (0–2)	0.242
BODE Index **§	1 (0–2)	0 (0–2)	0.191
Charlson Index **§	1 (1–1)	0 (0–1)	0.740
Emphysema (%) †§	67	57	0.424
Lung cancer stage (%)			
I	62	-	
II	9	-	
III	19	-	
IV	10	-	

* Data are presented as mean ± SD. ** Data are presented as median (25th–75th pc). ‡ Number of packs of cigarettes smoked per day × number of years smoking. BMI: body mass index; FEV_1_: forced expiratory volume in one second; FVC: forced vital capacity; % pred: percent predicted; PaO_2_: partial oxygen tension; K_CO_: transfer factor coefficient of the lung for carbon monoxide, which is DLCO; IC/TLC: inspiratory capacity to total lung capacity ratio; 6MWD: six-minute walking distance test. † Emphysema, diagnosed using CT scans. § This measure was available for 11 individuals with COPD and LC. *p*-value < 0.05 (in bold).

**Table 2:** Demographical and clinical characteristics of smoking COPD patients that did and did not develop lung cancer (LC) during follow-up monitoring, three years before diagnosis.

Variable	COPD with LC n = 21	COPD without LC n = 42	*p*-Value
Age *	60 ± 9	60 ± 9	-
Sex (male%)	85	85	-
Smoking habit (pack-year) ‡	65 ± 20	63 ± 27	0.554
BMI	28 ± 5	28 ± 4	0.662
FEV_1_ (L) *	2.20 ± 0.78	1.61 ± 0.56	0.004
FEV_1_ (% pred) *	76 ± 22	59 ± 22	0.007
FVC (% pred) *	102 ± 21	91 ± 22	0.055
FEV_1_/FVC (% pred) *	58 ± 12	51 ± 13	0.032
PaO_2_ *§	74 ± 5	72 ± 10	0.457
K_CO_ *§	72 ± 16	86 ± 26	0.045
IC/TLC (%) *§	36 ± 8	36 ± 9	0.961
6MWD (mts) *§	532 ± 59	525 ± 88	0.767
Dyspnea mMRC **	0 (0–12)	1 (0–2)	0.242
BODE Index **§	1 (0–1)	1(0–2)	0.645
Charlson Index **§	1 (1–2)	0(0–1)	0.058

* Data are presented as mean ± SD. ** Data are presented as median (25th–75th pc). ‡ Number of packs of cigarettes smoked per day × number of years smoking. BMI: body mass index; FEV_1_: forced expiratory volume in one second; FVC: forced vital capacity; % pred: percent predicted; PaO_2_: partial oxygen tension; K_CO_: transfer factor coefficient of the lung for carbon monoxide, which is DL_CO_; IC/TLC: inspiratory capacity to total lung capacity ratio; 6MWD: six-minute walking distance test. § 11 COPD-with-LC individuals were analyzed for these variables. *p*-value < 0.05.

**Table 3:** Association between the studied biomarkers and lung cancer risk in smokers with COPD three years before LC diagnosis.

Variables	HR	95% CI	*p*-Value
T/S1	7.47	2.16–25.83	**0.001**
8-OHdG	2.13	1.20–3.99	**0.01**

Multivariate analysis adjusted by the following covariates: smoking (pack years), BMI (body mass index), and emphysema (score based on CT scans); HR, hazard ratio. A *p*-value < 0.05 was considered significant. *p*-value < 0.05 (in bold).

**Table 4:** Distribution of *TERT*, *RTEL1*, and *NAF1* genetic variants in COPD patients with LC and COPD cases without LC as controls included in this study.

SNVs	COPD Controls (n = 60)	COPD with LC (n = 30)	OR ^d^ (95% CI)	*p*-Value
*TERT* rs2736100				
^a^ C/C	18 (39.1%)	8 (27.6%)	Ref	0.59
A/C	17 (37%)	13 (44.8%)	1.72 (0.57–5.18)	
A/A	11 (23.9%)	8 (27.6%)	1.64 (0.48–5.62)	
^b^ C/C	18 (39.1%)	8 (27.6%)	Ref	0.3
A/C-A/A	28 (60.9%)	21 (72.4%)	1.69 (0.62–4.62)	
^c^ C/C-A/C	35 (76.1%)	21 (72.4%)	Ref	0.72
A/A	11 (23.9%)	8 (27.6%)	1.21 (0.42–3.50)	
*RTEL1* rs755017				
A/A	35 (76.1%)	24 (80%)	Ref	0.69
A/G	11 (23.9%)	6 (20%)	0.80 (0.26–2.44)	
*NAF1* rs7675998				
^a^ G/G	30 (68.2%)	18 (60%)	1.00	0.49
A/G	11 (25%)	11 (36.7%)	1.67 (0.60–4.62)	
A/A	3 (6.8%)	1 (3.3%)	0.56 (0.05–5.75)	
^b^ G/G	30 (68.2%)	18 (60%)	1.00	0.47
A/G-A/A	14 (31.8%)	12 (40%)	1.43 (0.54–3.76)	
^c^ G/G-A/G	41 (93.2%)	29 (96.7%)	1.00	0.5
A/A	3 (6.8%)	1 (3.3%)	0.47 (0.05–4.76)	

**^a^** Co-dominant model; **^b^** Dominant model; **^c^** Recessive model of inheritance. OR, odds ratio; CI, confidence interval; FDR, false discovery rate. Benjamini–Hochberg method. **^d^** Adjusted for age, sex, and smoking status.

## Data Availability

The datasets supporting the conclusions of this article are included within the article and its Supplementary Materials.

## Supplementary Materials

The following supporting information can be downloaded at: https://www.mdpi.com/article/10.3390/antiox13020156/s1, Table S1: Comparison of clinical characteristics of smoking patients with COPD and lung cancer (LC) at the time of LC diagnosis and three years before.
