# Supplementary material for: Oxidative Damage and Telomere Length as Markers of Lung Cancer Development among Chronic Obstructive Pulmonary Disease (COPD) Smokers
Source: Antioxidants (Basel). 2024 Jan 26;13(2):156. doi: 10.3390/antiox13020156 (PMC10886051; doi:10.3390/antiox13020156)
Supplement: Supplementary file 1 [file antioxidants-13-00156-s001.zip › antioxidants-2824334-supplementary.pdf]

Supplementary material

Table S1. Comparison of clinical characteristics of smoker patients with COPD and lung cancer (LC) at the LC diagnosis time and three years before.

**Variable**	**COPD with LC n=21 At diagnosis**	**COPD with LC n=21 3-yrs before**	***p-value***
BMI*	28±5	27±4	0.148
FEV_1 (L)*	2.11±0.77	2.18±0.78	0.090
FEV_1 (% pred)*	75±24	76±22	0.745
FVC (% pred)*	103±23	102±21	0.809
FEV_1/FVC (% pred)*	57±12	58±12	0.009
PaO_2*§	73±4	74±5	0.741
KCO*§	63±19	72±16	0.021
IC/TLC (%)*§	33±8	36±9	0.100
6MWD (mts)* §	484±123	532±59	0.085
Dyspnea mMRC**	0 (0 - 1)	0 (0 - 1)	0.090
BODE index **§	1 (0 - 2)	1 (0 - 1)	0.044
Charlson index**§	1 (0 - 1)	1 (1 - 2)	0.004
Relative T/S**	1.16±0.31	1.29±0.36	0.228
TL absolute	13177±3264	14511±3697	0.228

*Data are presented as mean ±SD. ** Data are presented as median (25th-75^{th}pc). ‡Number of packs of cigarettes smoked per day x number of years smoking. T/S ratio: relative telomere length; TL: telomere length; BMI: body mass index; FEV_1: forced expiratory volume in one second; FVC: forced vital capacity; % pred: per cent predicted; PaO_2: partial oxygen tension; K_{CO}: transfer factor coefficient of the lung for carbon monoxide, which is DL_{CO}; IC/TLC: inspiratory capacity to total lung capacity ratio; 6MWD: six minutes walking distance test. §11 COPD with LC individuals were analysed for these variables.
